# Clinical and Economic Impact of Implementing OVIVA Criteria on Patients With Bone and Joint Infections in Outpatient Parenteral Antimicrobial Therapy

**DOI:** 10.1093/cid/ciz991

**Published:** 2019-10-11

**Authors:** Michael Marks, Lucy C K Bell, Imogen Jones, Tommy Rampling, Katharina Kranzer, Stephen Morris-Jones, Sarah Logan, Gabriele Pollara

**Affiliations:** 1 Division of Infection, University College London Hospitals, London, United Kingdom; 2 Clinical Research Department, London School of Hygiene and Tropical Medicine, London, United Kingdom; 3 Department of Clinical Microbiology, University College London Hospitals, London, United Kingdom; 4 Division of Infection and Immunity, University College London, London United Kingdom

**Keywords:** OVIVA, OPAT, IV antibiotics

## Abstract

The OVIVA study demonstrated noninferiority for managing bone and joint infections (BJIs) with oral antibiotics. We report that 79.7% of OPAT patients being treated for BJIs at our center would be eligible for oral antibiotics, saving a median (IQR) 19.5 IV-antibiotic days (8.5–37) and GBP 1234 (569–2594) per patient.

Bone and joint infections (BJIs) are conventionally managed with up to 6-week courses of antibiotic therapy [1–3]. Alongside appropriate surgical intervention, intravenous (IV) antibiotics are commonly recommended to maximize tissue penetration [1, 2]. Delivery of IV therapy is increasingly provided by outpatient parenteral antimicrobial therapy (OPAT) services enabling earlier discharge from the hospital [4]. Although OPAT is effective and popular [4], it still carries risks of adverse events due to requirements for IV catheters [5] as well as the IV antibiotics being administered [6]. Therefore, there has been great interest in the potential role of oral antibiotics in managing BJIs.

A multicenter, pragmatic, randomized controlled trial (Oral Versus IntraVenous Antibiotic treatment for bone and joint infections [OVIVA] study) revealed that switching from IV to oral antibiotic therapy within 1 week of commencing treatment for BJI was non-inferior with respect to treatment failure after 1 year of follow-up compared with 6 weeks of IV antibiotics to treat a wide range of BJIs [7]. The study extrapolated reductions in IV antibiotic use and cost savings [7]. These findings are potentially paradigm shifting, but the impact of implementing such a change on real-world practice remains unknown. We used the OVIVA study criteria to infer eligibility of patients with BJIs in our OPAT service for oral antibiotic regimens and assess possible cost savings [5, 8].

## METHODS

### Patient Cohort and Data Extraction

We utilized data collected prospectively into the OPAT database at University College London Hospitals (UCLH) National Health Service Foundation Trust [5, 8]. We included all patients with a BJI treated via OPAT between January 2015 and October 2018. We did not include patients with BJI managed outside of OPAT. Microbiological results were extracted from the laboratory information system.

The study was approved by the Audit and Research Committee at the Hospital for Tropical Diseases, UCLH, which stated that, as this was a retrospective review of routine clinical data, formal ethical approval was not required.

### Case Review and Analysis

Suitability for an oral regimen was determined by a consultant microbiologist not involved with the OPAT service (K. K.). Clinical diagnosis, microbiological data, and allergies were reviewed to ascertain if an effective oral antibiotic regimen was available to replace the IV regimen. In the absence of microbiological investigations, we constructed empirical oral antibiotic combinations that included clindamycin, ciprofloxacin, or rifampicin, aiming to cover the most common syndrome-associated causative organisms (Supplementary Table 2). We closely mirrored enrollment into the OVIVA study, considering patients not to be suitable for an oral regimen if they met any of the stated trial exclusion criteria [7]. We classified patients’ surgical interventions using the categories presented in the OVIVA trial [7]. We considered ineligible a subset of patients with a coagulase negative staphylococcal infection (CoNS) where linezolid and/or chloramphenicol were the only oral options, based on toxicities related to prolonged use of these drugs.

### Statistical Analysis

We estimated reduction in days of IV treatment and economic savings by following the OVIVA protocol of IV therapy being administered for 1 week prior to oral antibiotic switch. We deliver OPAT via community nursing services, attendance at hospital clinics, or self-administration, depending on patient requirements. We considered the median cost of delivering these services, the cost of therapeutic drug monitoring, and a one-off cost of IV catheter insertion (Supplementary Table 1). We did not include costs of other equipment, such as flushes, nor did we assign any costs when patients self-administered OPAT. Daily antibiotic costs were derived from our hospital pharmacy list pricing for both IV and oral antibiotics (Supplementary Table 1). We did not include the cost of a weekly outpatient review, as we assumed this would be indicated irrespective of whether the patient was receiving IV or oral antibiotics. As many patients in our service already receive fewer than 6 weeks of IV antibiotics, we also estimated hypothetical savings if patients had all, in fact, received 6 weeks of IV therapy. Statistical analysis was performed using R version 3.4.2.

## RESULTS

We identified a total of 133 patients treated for BJIs through our OPAT service, comprising approximately one-quarter of all our OPAT patients [5]. Non-vertebral, native osteomyelitis was the most common type of BJI, with a range of other native and prosthetic joint infections also being treated (Table 1). The majority of patients (74%) underwent a therapeutic or diagnostic surgical procedure (Table 1). Oral antibiotic treatment, in line with OVIVA, was considered appropriate for 106 (79.7%) patients. The most common reason patients were not eligible was the absence of a suitable oral agent on antibiotic susceptibility testing (n = 14, 10.5%), including for 8 of 18 (44.4%) CoNS infections in our cohort (Table 1).

**Table 1.** Characteristics of All Outpatient Parenteral Antimicrobial Therapy Patients Being Treated for Bone and Joint Infections, Categorized According to Eligibility for Receiving Treatment With Oral Antibiotic Regimens

	Overall (N = 133)	Eligible for Oral Antibiotics (n = 106)	Not Eligible for Oral Antibiotics (n = 27)
Male, n (%)	92 (68.1)	74 (69.8)	18 (66.7)
Age in years (IQR)	62 (46–71)	62 (42.5–72)	63 (49–68)
Diagnosis, n			
Discitis/vertebral osteomyelitis	25	23	4
Diabetic foot	8	6	2
Osteomyelitis (non-vertebral)	52	37	13
PJI			
Knee	21	19	2
Hip	20	16	4
Other	7	5	2
Surgical interventions, n (%)			
Debridement, antibiotics, and implant retention	13 (9.8)	9 (8.5)	4 (14.8)
No implant or device present; debridement of chronic osteomyelitis not performed	17 (12.8)	14 (13.2)	3 (11.1)
No implant or device present; debridement of chronic osteomyelitis performed	22 (16.5)	17 (16)	5(18.5)
PJI removed	14 (10.5)	12 (11.3)	2 (7.4)
PJI, 1-stage revision	27 (20.3)	19 (17.9)	8 (29.6)
Removal of orthopedic device for infection	16 (12)	15 (14.2)	1 (3.7)
Surgery for discitis, spinal osteomyelitis, or epidural abscess; debridement not performed	22 (16.5)	18 (17.0)	4 (14.8)
Surgery for discitis, spinal osteomyelitis, or epidural abscess; debridement performed	2 (1.5)	2 (2.2)	0 (0)
Reasons could not receive oral agent, n (%)			
Antibiogram of causative organism did not offer viable oral regimen	N/A	N/A	14 (51.9)
*Staphylococcus aureus* bacteremia within the preceding 30 days	N/A	N/A	7 (25.9)
Fungal BJI	N/A	N/A	4 (14.8)
Allergy to the only oral antibiotic regimens possible	N/A	N/A	2 (7.4)
Duration of total IV therapy, median (IQR),^a^ days	29 (18–45)	26.5 (15.5–44)	43 (29.5–58.5)
Duration of IV therapy through OPAT, median (IQR), days	20 (12–34)	17 (11–32)	26 (19–42.5)
Extrapolated reduced length of IV antibiotic therapy per patient, median (IQR),^b^ days	N/A	19.5 (8.5–37)	0
Extrapolated cost savings per patient, median (IQR),^b^ GBP	N/A	1234 (569–2594)	0
Extrapolated daily cost savings per patient, median (IQR),^b^ GBP	N/A	63 (29–133)	0

Abbreviations: BJI, bone and joint infection; GBP, pounds sterling; IQR, interquartile range; IV, intravenous; N/A, not applicable; OPAT, outpatient parenteral antimicrobial therapy; PJI, prosthetic joint infection

^a^The total duration of IV therapy considering both inpatient and OPAT IV therapy combined.

^b^These estimates were derived from a scenario of patients receiving 1 week of IV antibiotics prior to changing to an appropriate oral antibiotic regimen that matched the findings of their microbiological investigations.

Within the “oral therapy eligible” group (n = 106), the 2 most commonly used IV drugs were ceftriaxone (n = 56, 52.9%) and teicoplanin (n = 53, 50%), while carbapenems were used in a minority of patients (n = 10, 9.5%) (Supplementary Table 2). Overall, a significant proportion of these patients also received rifampicin (n = 43, 40.6%) or ciprofloxacin (n = 39, 36.8%). Of 106 “oral therapy eligible” patients, 85 (80.2%) had microbiological data available to guide antibiotic choice and 21 (19.8%) did not. A variety of hypothetical oral regimens could be constructed; rifampicin and ciprofloxacin (n = 43) was the most common. In the “oral therapy eligible” group, the median duration of IV therapy was 26.5 (interquartile range [IQR], 15.5–44) days compared with patients who did not meet the criteria for oral therapy, who received a median of 43 (IQR, 29.5–58) days (*P* = .02). Both groups received the majority of IV therapy through the OPAT service (Table 1).

In our 106 “oral therapy eligible” patients, we estimated a total of 2589 days of IV treatment saved, with a median saving per patient of 19.5 (IQR, 8.5–37) IV antibiotic days. Total cost saved was estimated at pounds sterling (GBP) 185 788 (median, GBP 1234; IQR, GBP 569–2594 per patient). If all patients had, in fact, received 6 weeks of IV therapy, rather than a median of 26.5 days, then the estimated median cost saving was GBP 2950 (IQR, GBP 1725–2950).

## DISCUSSION

OVIVA provided strong evidence that BJIs could be treated with appropriate oral antibiotic regimens, but most patients were recruited from 2 specialist BJI centers [7]. Making use of a well-defined and prospectively recorded cohort of patients receiving OPAT [5, 8], we provide the first real-world assessment that implementation of comparable criteria rendered the majority (80%) of our BJI cohort eligible for oral antibiotic treatment. We had elected not to participate in OVIVA as our practice at the time of recruitment was shorter than 6 weeks of IV therapy, reflected in median IV therapy duration of 29 (IQR, 18–45) days. In addition, inpatients discharged directly on oral antibiotics following short IV antibiotic courses were not included in this cohort as they were not referred to the OPAT service. Therefore, IV antibiotic and cost savings may be greater in centers routinely using longer courses of IV therapy. Our estimation suggests that centers routinely using 6 weeks of IV antibiotics might anticipate savings closer to GBP 2000–3000 per patient.

We estimated that increased use of oral antibiotics would result in significant reductions in IV antibiotic use, with ensuing reduced risks associated with IV catheters [5], as well as substantial cost savings. Nevertheless, we acknowledge that oral antibiotics still carry risks of adverse events that need monitoring [9]. Indeed, our cost analysis excluded any reductions in visits to the outpatient department for patients receiving oral antibiotics for this reason. We previously demonstrated that both drug- and IV catheter–associated adverse events are uncommon in our cohort [5] and the OVIVA study did not demonstrate differences in the incidence of serious adverse events between the 2 arms [7]. Therefore, we did not factor into our cost calculations savings associated with reductions in adverse events related to IV antibiotics. However, the frequency of adverse events related to IV antibiotics in other cohorts may be greater, and IV antibiotic use may be associated with other unrecorded costs, such as complications with IV drug administration in the community requiring hospital review. We acknowledge that these factors may render our cost calculations an underestimate of the true savings of oral antibiotic therapy.

Our findings were derived from a single OPAT center, although data were collected prospectively as part of routine clinical care and BJIs made up a comparable proportion of OPAT cases as elsewhere [10]. Despite independent review, decisions on oral regimens could not account for patient-specific factors, including unanticipated drug intolerances, and clinician-specific opinions, such as the suitability of β-lactams for BJIs [11]. We may have overestimated eligibility for oral regimens if a requirement for IV therapy was not recorded, such as drug interactions and oral drug intolerance. We considered patients eligible for oral therapy when there was no microbiological diagnosis on sampling (19.8% of our cohort), which was comparable to patients in the OVIVA study (20%) [7].

In conclusion, we present the first assessment of the potential impact of implementing the OVIVA findings in a real-world OPAT setting. The majority of patients could have been placed on an oral regimen with significant cost savings. The challenge remains to identify the optimal oral antibiotic regimens and durations to effectively deliver excellent clinical outcomes in the treatment of BJIs [12].

## Supplementary Data

Supplementary materials are available at *Clinical Infectious Diseases* online. Consisting of data provided by the authors to benefit the reader, the posted materials are not copyedited and are the sole responsibility of the authors, so questions or comments should be addressed to the corresponding author.

ciz991_suppl_Supplementary_Table

## Notes

***Acknowledgments.*** The authors thank the dedicated group of OPAT nurses who have compiled the prospective data over the duration of this study and beyond.

***Disclaimer.*** The funding agencies had no role in study design, data collection and analysis, decision to publish, or preparation of the manuscript.

***Financial support.*** This work was supported by the Wellcome Trust (grant numbers WT101766 to G. P. and WT102807 to M. M.). All authors were supported by the University College London Hospitals Comprehensive Biomedical Research Centre Infection Theme.

***Potential conflicts of interest.*** The authors: No reported conflicts of interest. All authors have submitted the ICMJE Form for Disclosure of Potential Conflicts of Interest.
